# How do American and British Nonsmokers Value Secondhand Smoke Health Risks?

**DOI:** 10.1007/s10935-023-00752-0

**Published:** 2023-11-24

**Authors:** Eleanya Nduka

**Affiliations:** Department of Economics, University of Warwick, Coventry, UK

**Keywords:** Secondhand smoke, Health risks, Welfare, WTP, WTA, Choice experiment, Contingent valuation, I18, I31

## Abstract

Despite concerted efforts to enforce smoke-free laws in various countries, nonsmokers, particularly women and children, continue to be exposed to daily secondhand smoke (SHS), resulting in significant health risks. While existing studies have assessed the health effects of numerous diseases, the quantification of SHS spillovers remains understudied. This research employs choice experiments and contingent valuation techniques to rigorously quantify the attributes of SHS health risks, with a specific emphasis on facilitating cross-country comparisons. Our investigation reveals that nonsmoking individuals in the United Kingdom exhibit an attitude of indifference towards a proposed policy offering increased disposable income as compensation for SHS exposure. Conversely, nonsmoking Americans express a contrary perspective. Furthermore, our study demonstrates that nonsmoking Americans attribute a higher value to SHS health risks compared to their British counterparts. Consequently, this research uncovers a hitherto unexplored dimension of health risk-related behaviors. These findings hold the potential to significantly contribute to the development of future smoke-free policies, offering valuable insights that can inform policy decisions and address the persistent challenges associated with SHS exposure, particularly among vulnerable populations.

## Introduction

A few countries have enacted various smoke-free laws to protect nonsmokers from exposure to secondhand smoke (SHS). Governments increase tobacco taxes to raise cigarette prices and ultimately discourage smoking. However, tobacco companies counteract this through price discounts. Such laws are helpful but do not eliminate SHS, leaving many nonsmokers at risk in some public and private places (van der Eijk & Porter, 2015). Restricting smoking in some public venues increases smoking in private places as smokers try to make up for the loss of certain outdoor smoking (Agee et al., 2001; WHO, 2023). Studies have shown that indoor exposure to SHS increased in recent years (Ciaccio et al., 2014; de Hollander and Melse, 2006; Yang et al., 2021), especially during COVID-19 lockdown (ASH, 2020; Yach, 2020).

One common feature of smoke-free laws in many countries is that they do not protect nonsmokers in private homes and other public places such as bus stops, building entrances, often-crowded city centers, and other outlets. There is also smoke drift from one apartment to another, especially in multi-unit housing (CDC, 2021b, 2021c). Thus, smoke-free laws are far from fulfilling Article 8 of the WHO Framework Convention on Tobacco Control (WHO, 2013, 2023).

The WHO promotes a smoke-free world because no level of exposure is safe, and using designated areas for smoking and other measures have not been effective in achieving clean air (WHO, 2013). Globally, about one in two children breathes in SHS, and 65,000 die as a result. In total, 1.2 million nonsmokers die prematurely each year (WHO, 2020). In addition, recent evidence shows that children exposed to SHS are vulnerable to sudden infant death syndrome, middle ear disease, and asthma. Also, adults exposed to SHS have a 20–30% risk of suffering stroke, lung cancer (20–30%), and coronary heart disease (25–30%) (CDC, 2020). Emotional distress has also been reported among those exposed to SHS (Bandiera, Richardson, Lee, He and Merikangas, 2011). Furthermore, it is worth noting that exposure to SHS increases the risk for COVID-19 infection (Mendez, Escobar, Encinas and Wojcicki, 2021) and other infectious diseases (Jiang et al., 2020; The Cancer Council, 2019).

Based on the smoking prevalence in the U.S. and U.K., approximately 23% of Americans aged 15 years and above are tobacco users, with 28% of males and 18% of females engaging in tobacco consumption (World Bank, 2023). Consequently, SHS exposure is responsible for over 41,000 deaths among nonsmokers annually. Among these fatalities, approximately 34,000 are attributed to heart disease, 8000 to stroke, and 7300 to lung cancer (CDC, 2021a). These figures underscore the profound health risks nonsmokers face due to SHS exposure and emphasize the urgent need for effective interventions to mitigate its adverse consequences.

In comparison, 15.4% of British aged 15 years and over use tobacco—17% of males and 14% of females (World Bank, 2023). Exposure to SHS accounts for more than 300,000 General Practice (GP) consultations annually with children, about 9,500 are admitted to hospitals, and 40 cot deaths are recorded (SFF, n.d.). In addition, SHS is responsible for around 10,700 deaths among nonsmokers each year (ASH, 2020).

While scientific evidence shows that SHS has enormous health risks, it is unclear how nonsmokers perceive and value these risks. Stated preference methods such as contingent valuation (Andersson et al., 2015; Hammitt & Haninger, 2010; Hollinghurst et al., 2016; Tubeuf et al., 2015), and choice experiments (Adamowicz et al., 2011; Gerard et al., 2003; Hole, 2008; Huang et al., 2018; Johnson et al., 2000) are widely used to value health risks where there is a lack of market data. These techniques involve creating a hypothetical market in which respondents make choices that involve trade-offs between health risks and a certain payoff (Andersson et al., 2019).

However, critical issues need to be considered in valuing spillovers such as SHS. These issues involve deciding whether to elicit willingness to pay (WTP) or willingness to accept (WTA). The choice depends on two factors: (1) the nature of the good and (2) property rights (Carson et al., 2001; Hammitt, 2002; Knetsch, 2007; Kim et al., 2015; Whittington et al., 2017).

Thus, when an individual has the right to a welfare improvement and yet does not obtain it, WTA is appropriate. On the other hand, the individual is expected to pay for the improvement if they are not entitled, in which case, WTP is elicited. The implication is that an individual who has the right to produce negative externalities should be incentivized if the government wants to curtail such activities.

Against this background, valuing SHS health risks is not straightforward because nonsmokers are entitled to clean air free from tobacco smoke (UNEP, 2019a, 2019b; WHO, 2005). At the same time, smokers have the right to smoke in non-smoke-free zones whether or not a nonsmoker is present. This study is the first to give insight into nonsmokers’ perceptions about SHS and quantify its welfare impacts, including the psychological aspect. As a result, it would give policymakers more insight into peoples’ views on this issue and contribute to smoke-free legislation.

The selection of the United States (U.S.) and the United Kingdom (U.K.) as our study focus is based on the intrinsic dissimilarity between their healthcare systems. While the U.S. operates a private healthcare system, the U.K. adopts a publicly-funded approach. This divergence prompts us to investigate how nonsmokers from these two countries value the health risks associated with secondhand smoke differently, drawing on insights from the theory of social preferences and social capital.

The theory of social preferences posits that individuals are not solely driven by self-interest but also exhibit concern for the welfare of others. Consequently, decision-making may extend beyond individual benefits to encompass collective well-being. Social capital, on the other hand, encapsulates the value derived from the interconnected networks of relationships among members of a given society, which fosters effective societal functioning. It comprises robust interpersonal ties, shared identities, mutual understanding, norms, values, trust, cooperation, and reciprocity (Charness & Rabin, 2002; Martikke, 2017; Portes, 1998).

In light of these conceptual frameworks, we posit that the U.S. nonsmokers, accustomed to a privately funded or out-of-pocket medical services system, may exhibit a higher valuation of secondhand smoke health risks compared to their British counterparts, who experience a publicly-funded healthcare system. The difference in healthcare financing mechanisms could influence individuals’ risk perception and response patterns, shaping their willingness to pay to prevent exposure and/or willingness to accept compensation.

In the context of the U.S. private healthcare system, where individuals bear a more direct financial responsibility for their medical expenses, the notion of social preferences may take a back seat, potentially leading to a stronger emphasis on personal well-being. In contrast, the U.K.’s publicly-funded healthcare system may foster a sense of shared responsibility and mutual support.

It is imperative to acknowledge that this argument constitutes a theoretical framework and requires empirical investigation to substantiate the actual differences in how American and British nonsmokers value secondhand smoke health risks. Nevertheless, the exploration of such disparities has the potential to enhance our understanding of the intricate interplay between healthcare system structures, social dynamics, and risk valuation among nonsmokers in these two nations. By shedding light on these aspects, our study endeavors to contribute valuable insights to public health policy formulation, encouraging tailored interventions that address the specific needs and behaviors of nonsmokers in each context.

## Literature Review

Although there are no studies directly related to ours, a handful of studies have used stated preference (SP) techniques to elicit willingness to pay to reduce health risks or undergo a diagnostic test. We review these studies to give insights into the types of health-related diseases researchers have evaluated. The WTP values reported in the literature differ significantly depending on the elicitation format and period of study (affected by inflation and exchange rate) and the type of healthcare system practiced in the place of study. Thus, readers should be cautious in comparing WTP values. Furthermore, while some reported median, others presented mean WTP, which is usually greater than the median value due to outliers.

For example, O’Brien and Viramontes (1994) used contingent valuation (CV) to elicit WTP for a possible 99% chance of preventing chronic lung disease and a 1% likelihood of death in Canada. The participants were willing to pay CAN$113 per month to avoid the disease, while Johnson et al. (2000) applied choice experiments (CE) to value the attributes of respiratory and cardiovascular diseases, such as activity restriction, episode duration, and symptom. The estimated marginal WTP ranged from CAN$100 to CAN$225. Similarly, Krupnick et al. (2002) elicited willingness to pay for a reduction in mortality risk. The mean WTP was CAN$417, and the value for a statistical life (VSL) was CAN$1.2–$3.8 million. Adamowicz et al. (2011) applied CV and CE to value reductions in cancer and microbial diseases from drinking water. The attributes were microbial illness and death, cancer illness and death. The marginal WTP for fewer microbial and cancer deaths were CAN$12.61 and CAN$10.43, whereas the marginal WTP for reductions in cancer morbidity and microbial morbidity risks were CAN$2.43 and CAN$0.02. However, CV results show that the mean WTP for reductions in both risks was CAN$294. The VSLs for microbial and cancer deaths were CAN$16-$20 million and CAN$14–$17 million, respectively.

Arana and León (2006) estimated the WTP to reduce the risk of an episode of respiratory disease in Spain. The participants’ WTP was €41.70. Hammitt and Haninger (2010) estimated WTP to reduce fatal diseases and trauma such as cancer and other chronic diseases to children and adults. The VSL for children was $12–$15 million and US$6–$10 million for adults. Similarly, Andersson et al. (2015) studied Swedish respondents’ willingness to pay to reduce foodborne morbidity risks. The WTP was US$14,000-$17,700, and the VSL in a split-sample were US$710 million and US$695 million (Andersson,Hole and Svensson, 2016).

A few studies focus on China’s air quality, such as Hammitt and Zhou (2006), who estimated air pollution’s health risks such as cold, chronic bronchitis, and fatality. The median WTP was between US$3 and US$6 to prevent the risk of catching a cold, and US$500–$1000 for chronic bronchitis. The VSL was US$4000–$17,000. This is small because the authors used the median value instead of the mean, which is about thirteen times higher. Also, this result is about 10–1000 times smaller than the US’s and Taiwan’s at the exchange rate during the study. Likewise, Guo et al. (2006) investigated a similar problem (reduction in the risks of asthma and mortality) by considering three scenarios: when the policy aimed at reducing health risks is publicly or privately provided; how WTP changes with a change in the magnitude of the health risk; and the degree of sensitiveness of the WTP in the first scenario. The estimated median WTP for a reduction in asthma was US$1,711.

However, in an earlier study, Peng and Tian (2003) reported US$2717 as the WTP for reducing the risk of asthma. Huang et al. (2018) quantified the attributes of air pollution health risks, such as cold, respiratory, and cardiovascular diseases, and the probability of dying from those diseases. The VSL was US$774,000. Generally, the WTP to avoid pollution-related diseases in China is high because exposure to industrial air pollution is a severe unavoidable problem in many Chinese megacities.

Others have focused on the U.K. For instance, Frew et al. (2001) investigated the WTP for two types of colorectal cancer screening. Patients were willing to pay between £30 and £50. It was found that male respondents and those that had an experience of a health condition were willing to pay more. Also, Tubeuf et al. (2015) studied respondents’ willingness to pay for an inherited retinal disease test. Three scenarios were presented to participants: a test that would reveal the disease’s inheritance nature; a test that would provide information about the future’s visual function; and a new treatment that would improve patients’ condition. The mean WTP in those scenarios were £539, £1516, and £6895. In all the scenarios, patients were willing to undertake the tests. WTP decreased with the female respondents.

Similarly, Hollinghurst et al. (2016) examined whether patients were willing to undergo diagnostic testing for pancreatic, colorectal, and lung cancer. The first part of the survey used a binary question format to ask patients to show whether they would accept a test, and the second asked how much they were willing to pay. The results were conflicting because most of the respondents who answered yes to the first question were unwilling to pay. The WTP for a chest X-ray was £365 if the risk was 10% high and £305 if it was 1% low. Using choice experiments, Hole (2008) quantified the attributes of a general practitioner’s choice, such as doctors’ knowledge of the patient, the doctor’s interpersonal manner, and thoroughness of the physical examination, flexibility of time of appointment, and appointment waiting days. The author included a cost variable even though these services are free at the point of delivery. The patients were willing to pay £1.71, £4.48, £2.53, £4.13, and £13.82 for those attributes.

The way people perceive health risks varies across healthcare systems. Also, perceptions about other people’s health-risk behaviors differ. For example, Shickle (1997) showed that when British respondents were asked to choose between giving treatment priority to a patient responsible for their illness and another who cannot be blamed, an overwhelming 74% of the respondents chose a nonsmoker over a smoker. Further, 80% of the respondents chose a patient with low alcohol consumption over a heavy drinker; 60% favored giving life-saving treatment to a patient with an inherited disease over an identical patient with diet-related disease. Similarly, Miraldo et al. (2014) investigated respondents’ behaviors toward the National Health Service (NHS), bearing the costs of risky behaviors such as smoking, alcohol consumption, sedentary life, unhealthy diet, and overeating. Half of the respondents agreed that the NHS should cover risky behaviors such as smoking, sedentary life, and heavy drinking. Those who engage in those behaviors were more likely to favor the NHS covering those costs. Further, they were more likely to support covering other people’s risky behaviors too.

Also, in the Netherlands, the respondents showed low solidarity with smokers and overweight people (Bonnie,van den Akker,van Steenkiste and Vos, 2010). Likewise, Van der Star and Van den Berg (2011) estimated Dutch respondents’ WTP to cover health-risk behavior such as smoking and non-lifestyle chronic disease in the primary health insurance package. About 80% of the respondents voted for a flat insurance premium regardless of healthcare utilization. The respondents were willing to pay €11.29 to include smoking behavior in the package compared to €42.39 for a chronic disease independent of their lifestyle.

In the U.S., Berk et al. (2006) reported that 52% of the respondents favored mandatory health insurance, 87% believed that health premiums should be flat and not vary with the health status of patients, 60% believed that people who engage in risky health behaviors such as smoking should pay higher premiums. About two-thirds of the respondents wanted the healthcare system to be publicly-funded. Jonas et al. (2010) elicited willingness to pay to avoid discomfort and time related to colposcopy. Participants were willing to pay US$263 to avoid those situations. On the other hand, using the 1991 National Maternal and Infant Health Survey (NMIHS), Agee et al. (2001) estimated smokers’ valuation of own and child’s health. The results suggest that smokers were willing to pay US$494.48 for a 10% increase in their child’s health annually. At the same time, they were willing to pay US$10.12 for a 1% reduction in their child’s exposure to ETS. However, the respondents were willing to pay US$198.29 for a 10% increase in their health. It was reported that WTP increased with the frequency of smoking and child exposure. Thus, smokers value their child’s health two times more than theirs. Neumann et al. (2012) studied respondents’ willingness to pay for Alzheimer’s, prostate cancer, arthritis, and breast cancer diagnostic tests. The median WTP was US$109–$263. Further, WTP declined with the female respondents.

First, prior research in this domain has predominantly focused on examining how individuals value the health risks of their behaviors. Surprisingly, the effects of other individuals’ health-risk behaviors on public health have been largely overlooked. By directing our investigation toward understanding how nonsmokers value the health risks associated with secondhand smoke and considering its impact on the wider public, our study brings attention to this novel behavioral aspect that has yet to be explored in the literature.

Second, a cross-country comparison is imperative to discern potential disparities in the valuation of secondhand smoke health risks between American and British nonsmokers. By juxtaposing the privately-funded healthcare system in the United States and the publicly-funded healthcare system in the United Kingdom, our study endeavors to fill this gap and shed light on how different healthcare financing structures may influence the perception and valuation of secondhand smoke health risks.

As a result, this research intends to unravel unique insights into the behavioral aspects of health-risk valuation concerning secondhand smoke, particularly concerning the consideration of its impact on public health and the comparative analysis across two countries with distinct healthcare systems. By addressing these unexplored dimensions, our study aims to contribute valuable knowledge to the existing literature and provide a more comprehensive understanding of how individuals from different healthcare contexts value the health risks associated with secondhand smoke exposure.

## Methodology

### Overview

We estimate both respondents’ willingness to accept  health harm compensation due to SHS and willingness to pay  to prevent exposure. Here, WTA is the amount of money that will keep an individual on a higher indifference curve (or the amount of money required to compensate the individual for health harms or losses) but on the same health risk level ($\hbox {R}_{0}$). Conversely, WTP is the amount that will be taken from the individual for a change from the status quo risk level ($\hbox {R}_{0}$) to an improved health level ($\hbox {R}_{1}$), while they are as well off as before.

We show the framework as follows: Assume an agent *n* with wealth $w_{0}$ and status quo health risk $R_{0}$. The utility functions of *n* are given as

1 $$\begin{aligned}{} & {} u(R^{n}_{0}, w^{n}_{0}) = u(R^{n}_{1}, w^{n}_{0}-WTP^{n}) \end{aligned}$$

2 $$\begin{aligned}{} & {} u(R^{n}_{0}, w^{n}_{0} + WTA^{n}) = u(R^{n}_{0}, w^{n}_{1}) \end{aligned}$$

where *WTP* is the amount of money *n* is willing to pay to prevent exposure to SHS, *WTA* is the amount *n* is willing to accept as compensation for health losses or harms due to SHS, and $R_{1}$ is an improved health profile.

### Contingent Valuation

We asked respondents to imagine a policy that would change their current level of SHS exposure. The payment vehicle was a one-off increment in their 1-year income tax. The question asked them to state the minimum and maximum WTP to prevent SHS exposure. It is a common practice to elicit maximum WTP only, but we included the minimum to ensure that zero responses in both cases signify a protest. We provided a cheap talk that reminded respondents to take the scenario as real and to be honest.

### Survey and Data Collection

Besides the first part that provided a brief introduction of the study to respondents and the second that contained the University of Exeter’s generic consent questions, the survey had five blocks.

In part one, we asked questions relating to respondents’ knowledge and perceptions  about SHS. Part two contained a few questions about their health status. Part three had WTP elicitation questions, including a question that asked respondents who indicated a zero WTP to state their reasons. Those who indicated a positive value were also asked to show how sure they were to pay the amount in reality on a ten-point Likert scale, ranging from not sure to very sure. In block four, we presented sixteen choice sets in two blocks of eight each. Respondents were randomly assigned these evenly. We asked a few socio-demographic questions in part five, including two debriefing questions that elicited respondents’ general views about SHS and the survey. The study terminated with a further explanation about SHS and how respondents can access the study’s findings in the future.

Participants were recruited and remunerated through the Prolific platform, a polling consultancy based in the United Kingdom. The sample consisted of individuals aged 18 years and above residing in the United States and the United Kingdom. Non-smokers were exclusively selected as participants in both countries, with an additional criterion for U.S. respondents requiring possession of health insurance. Prior to the primary survey, two pilot studies were conducted, encompassing both U.S. and U.K. populations. Subsequent to these preliminary investigations, adjustments to the questionnaire were made, involving the inclusion or removal of specific questions. A total of approximately 600 participants were surveyed in each country. The data collection period extended from September 14, 2020, to January 9, 2021. Following meticulous data cleaning procedures, 541 and 554 choice experiment responses from the U.S. and U.K. were employed in the estimation process, resulting in 8,656 and 8,864 observations, respectively. Notably, zero responses from the contingent valuation (CV) data were excluded, leading to the utilization of 364 and 363 observations from the U.S. and U.K., respectively.

### The Choice Experimental Design

Table 1 contains the attributes and their levels. The choice of attributes and levels were informed by extensive literature review and pilot studies. The attributes relate to sufficient evidence of health effects of SHS to adults, such as stroke, lung cancer, and coronary heart disease (see CDC, 2020, The Tobacco Atlas, 2021). Other attributes used are emotional distress and monetary payoffs (reduction in health insurance premium or tax as the case may be).

These attributes and their levels in the full factorial design yield 512 $(2 \times 4^4)$ different alternatives. These would be $512 \times 511/2 = 130,816$ pairs of choice sets, which will be too much for each respondent to complete. Thus, we constructed sixteen choice sets blocked into two pairs of eight choices using the D-efficient design. Without priors, and following the best practice guidelines, we set the attributes coefficients at zero (see Hole, 2008, 2017, Lancsar et al., 2017).

First, we designed the efficient experiment in Stata. Second, we transformed it into an Excel spreadsheet. Third, we wrote a Stata command in advanced format TXT files and constructed the CE tables using a hypertext markup language (HTML). Fourth, we randomized the order in which the choice sets were presented to participants within and across blocks using the advanced randomization option in Qualtrics. Finally, we wrote another Stata code that automatically transformed the collected data and made it ready for use (see Weber, 2019).

**Fig. 1:**
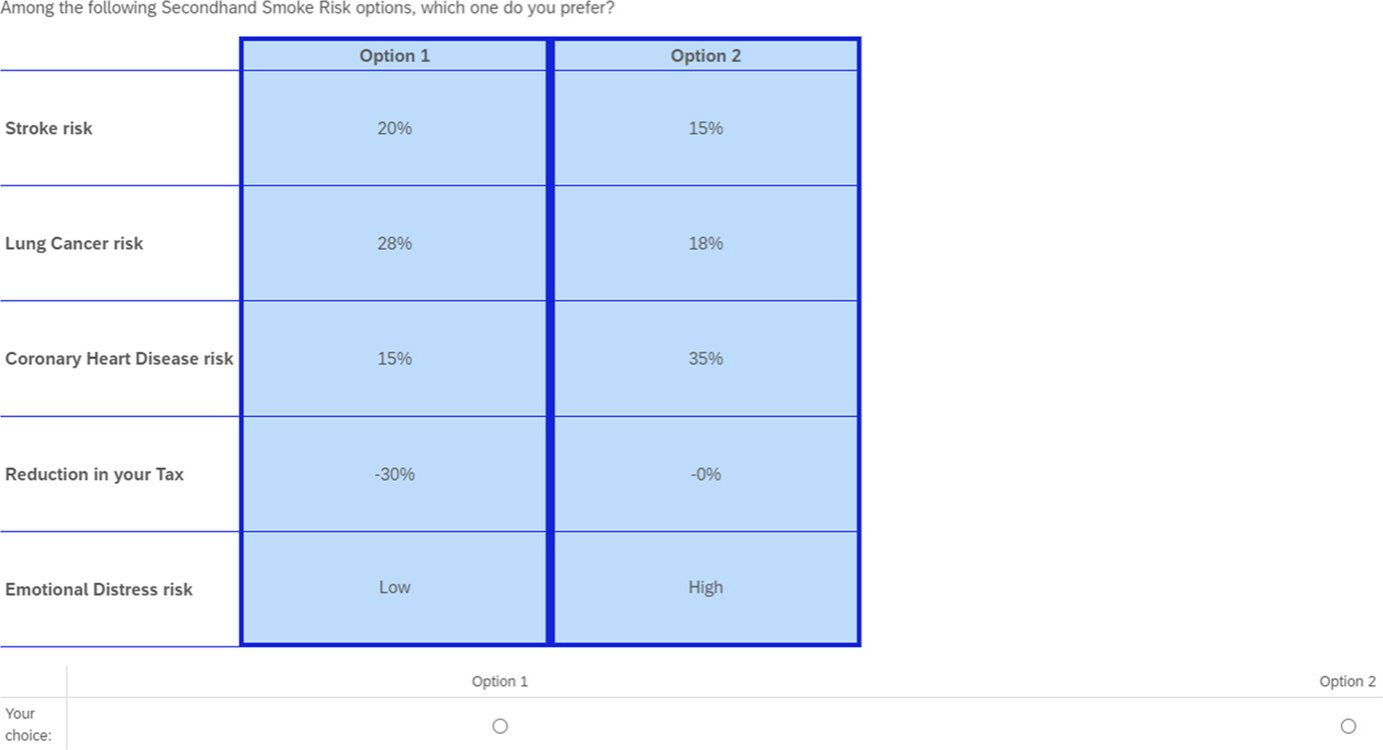
Example of choice task

The choice experiment scenario asked participants to imagine that they had to choose between two bundles of potential health risk levels due to SHS exposure, including a monetary payoff. Respondents were given a general introduction about the sources, effects, and meaning of each disease associated with SHS. Also, a brief country-specific statistics on the risk levels of the respective diseases were provided. This was to ensure that respondents focus on the subject. We provided a question that asked respondents to rank the attributes to their order of dislike before proceeding with the choice tasks. Figure 1 represents one of the choice sets presented to participants from the United Kingdom during the study.

**Table 1:** SHS risk attributes and levels

Attribute	Levels
Stroke risk	10%, 15%, 20%, 25%
Lung cancer risk	8%, 18%, 28%, 38%
Coronary heart disease risk	5%, 15%, 25%, 35%
Emotional distress risk	Low, high
Reduction in health insurance premiums (tax)*	0%, 10%, 20%, 30%

*We used health insurance premiums for U.S. participants and tax for U.K. respondents

### CV Models

One variant of CV uses open-ended questions that ask respondents to state their maximum WTP for an improvement in health conditions (see Contu & Mourato, 2020; Donaldson et al., 1998, Jonas et al., 2010). It is common to use ordinary least squares (OLS) regression to analyze the data in such a situation. However, this becomes problematic if the data set contains a substantial amount of zeros (Donaldson, Jones, Mapp and Olson, 1998) because of protest against paying for others’ health-risk behavior. It may be advisable to exclude the protest responses (Adamowicz et al., 2011; Johnston et al., 2017). The OLS regression is given by

3 $$\begin{aligned} \begin{aligned} \text{WTP}_{n}= \alpha + X^{\prime }_{n}\beta + \epsilon _{n} \end{aligned} \end{aligned}$$

where ${WTP}_{n}$ is the willingness to pay for respondent *n*, $X_{n}$ is a vector of the explanatory variables, $\beta$ is the vector of coefficients, $\epsilon _{n}$ is the error term. Equation 3 is estimated by minimizing $\sum _{n}\epsilon ^{2}_{n}$.

This model’s limitation is that it only gives the average relationship between the conditional mean of the dependent variable and a set of regressors, giving only a partial insight into the relationship (Cameron & Trivedi, 2010). Furthermore, even when the protest responses are removed from the estimation, there may still be some outliers, and because of the asymmetric distribution of the WTP, it is best practice to use the natural logarithm of WTP. Moreover, when such is done, if the smallest value of WTP is 1, it becomes 0.

The ensuing argument invokes the use of a censoring model such as the Tobit model. It assumes that the error term follows a censored normal distribution. The model is specified as:

4 $$\begin{aligned} \begin{aligned} E[WTP_{n}/X_{n}]&= \Phi (\beta ^{\prime }X_{n}/\sigma )\left( \beta ^{\prime }X_{n} + \frac{\sigma \phi (\beta ^{\prime }X_{n}/\sigma )}{\Phi (\beta ^{\prime }X_{n}/\sigma )}\right) \\&\quad = \Phi (\beta ^{\prime }X_{n}/\sigma )(\beta ^{\prime }X_{n}) + \sigma \phi (\beta ^{\prime }X_{n}/\sigma ) \end{aligned} \end{aligned}$$

where $\Phi$ and $\phi$ are the cumulative density function and standard normal density function, respectively, and $\sigma$ is the standard deviation of $\epsilon _{n}$. Here, the conditional mean function depends on the relationship between the dependent variable and a set of regressors and also on the probability that the dependent variable has a value that is greater than zero (Donaldson et al., 1998).

Another model favored over the OLS is quantile or median regression. It gives a complete view of the relationship between the dependent and independent variables at different quantiles in WTP distribution. Unlike the OLS regression, this model provides more robust results because it handles outliers efficiently. It is a semiparametric method, which does not make assumptions about the distribution of the error term (Cameron et al., 2005; Cameron & Trivedi, 2010). It is given by

5 $$\begin{aligned} \begin{aligned} WTP_{n}= \alpha + X^{\prime }_{n}\beta ^{q} + \epsilon _{n}^{q} \end{aligned} \end{aligned}$$

where $q \in (0,1)$ represents the quantile specified, the coefficients $\beta ^{q}$ are realized by minimizing the weighted sum of the absolute values of $\epsilon _{n}^{q}$.

To account for possible confounding effects, we include independent variables and specify the general form equation as:

6 $$\begin{gathered} WTP = f\left( {{\text{gender,~}}\;{\text{living}}\;{\text{~with}}\;{\text{~partner,}}\;{\text{~partner~}}\,{\text{smokes,~}}\;{\text{health~}}\;{\text{status}}} \right. \hfill \\ \quad \quad \quad \quad~\left. {{\text{secondhand}}\;{\text{~smoke~}}\;{\text{knowledge,~}}\;{\text{income,~}}\;{\text{etc}}} \right) \hfill \\ \end{gathered}$$

Hence, we are interested in the sign and statistical significance level of the independent variables on willingness to pay.

### CE Models

It is plausibly assumed that each decision-maker interprets utility in terms of attributes through a common functional form (Hensher et al., 2005). Suppose an individual *n* faces a set of alternative health scenarios denoted as *J* and $j = 1,\ldots ,J$. Let $U_{njt}$ represents the utility the *n* *th* individual derives from choosing the *j*th alternative in choice set *t*.

The individual’s utility is decomposed into representative (deterministic) and random parts. While the econometrician observes the representative part via estimation of model parameters, she is not aware of the random part (Train, 2009). The model can be specified in the additive form as:

7 $$\begin{aligned} U_{njt}{} & {} = \beta _{1}stroke_{njt} + \beta _{2}cancer_{njt} + \beta _{3}hrtdisease_{njt} \nonumber \\{} & {} \quad + \beta _{4}emodistress_{njt} + \beta _{5}payoff_{njt} + \epsilon _{njt} \end{aligned}$$

where $\beta _{1}$ to $\beta _{5}$ are the parameters to be estimated; $stroke_{njt}$ is stroke risk, $cancer_{njt}$ is lung cancer risk, $hrtdisease_{njt}$ is coronary heart disease risk, $emodistress_{njt}$ is the risk of having emotional distress, $payoff_{njt}$ is the reduction in health insurance premium or tax as the case may be, and $\epsilon _{njt}$ is the random error term.

Suppose we assume that the random terms are independently and identically distributed (IID) type I extreme value. In that case, this yields the conditional logit model of McFadden (1974).

8 $$\begin{aligned} P_{njt} = \frac{exp(\beta _{1}stroke_{njt} +\cdots + \beta _{5}payoff_{njt})}{\sum ^{J}_{j=1}exp(\beta _{1}stroke_{njt} +\cdots + \beta _{5}payoff_{njt)}} \end{aligned}$$

This model makes strong assumptions that the errors are IID, which leads to the second assumption of independence of irrelevant alternatives (IIA). This assumption states that the ratio of the probabilities of choosing any option over another $(\frac{P_{j}}{P_{i}})$ is not affected by the presence or absence of any other alternatives in the choice set. Thus, it treats respondents’ preferences and taste as homogeneous. However, in reality, the deterministic and random attributes of utility may depend on each other, and this correlation leads to the bias of the utility parameters.

As a result, more advanced models such as mixed logit (MXL) or random parameters logit (RPL), and generalized multinomial logit (G-MNL) are applied. The mixed logit model is highly flexible and guarantees a wide range of choices to specify individual-specific unobserved heterogeneity, although being fully parametric (Hensher & Greene, 2003). It overcomes the IIA assumption by treating the coefficients that enter the model as varying across individuals but being constant across choice occasions for each decision-maker (Train, 2009). In the mixed logit model, the unobserved part, $\epsilon _{nit}$ is independently and identically distributed extreme value over people and alternatives. The $\beta _{n}$ is a vector of coefficients [vector of parameter weights] representing individual-specific tastes with density $f(\beta /\theta )$, where $\theta$ represents, say, mean and covariance of the $\beta ^{\prime }$s in the population (Train, 2009). These conditional parameter estimates are strictly same-choice-specific parameters or the subpopulation’s mean that makes similar choices when faced with the same choice scenarios. It is an important distinction since it is impossible to establish every individual’s distinct set of estimates. Rather a mean estimate for the subpopulation who made the same set of choices is identified (Czajkowski et al., 2017; Hensher et al., 2005). It is worth noting that the MXL model collapses to the CL model if there is no unobserved heterogeneity. In which case, the CL can be reliable.

The probability that the decision-maker (*n*) makes a sequence of choices conditional on observing $\beta$ is the product of the logit formula as:

9 $$\begin{aligned} \begin{aligned} S_{n} = \int \prod ^{T}_{t=1}\prod ^{J}_{j=1}\left[ \frac{exp(\beta _{1}stroke_{njt} +\cdots + \beta _{5}payoff_{njt})}{\sum _{j}exp(\beta _{1}stroke_{njt} +\cdots + \beta _{5}payoff_{njt})}\right] ^{y_{njt}} f(\beta /\theta )d\beta \end{aligned} \end{aligned}$$

where $y_{njt}$ is equal to 1 if *j* alternative is chosen and to 0 otherwise, and $\beta = (\beta _{1}, \beta _{2}, \beta _{3}, \beta _{4}, \beta _{5})$. The econometrician determines $\beta$’s distribution through intuition and statistical tests. While parameters in Eq. 8 can be estimated using the maximum likelihood (ML) method, the integral in Eq. 9 can only be simulated. Despite the MXL model’s appealing qualities and its wide application, it is not free from criticisms. The model still assumes that the random error is IID.

The G-MNL models heterogeneity in taste as scale heterogeneity. This means that the scale of the error term is more significant for some respondents than others. In other words, the idiosyncratic error terms are more critical to some decision-makers than the observed attributes. Thus, it accounts for some respondents’ random behavior by treating the attributes coefficients as a continuous mixture of scaled normals (Fiebig et al., 2010; Lancsar et al., 2017). However, the MXL model with correlated coefficients may provide a better fit. It is best practice to estimate all the models and choose the best using AIC and BIC criteria.

For convenience, we will specify the G-MNL as (Lancsar et al., 2017):

10 $$\begin{aligned} U_{njt} = X^{\prime }_{njt}\beta _{n} + \epsilon _{njt} \end{aligned}$$

where $X^{\prime }_{njt}$ is a vector of respondent *n* observed attributes, and $\beta _{n}$ is a vector of respondent-specific coefficients.

11 $$\begin{aligned} \beta _{n} = \lambda _{n}\beta + \gamma \eta _{n} + (1 - \gamma )\lambda _{n}\eta _{n} \end{aligned}$$

No scale heterogeneity assume $\lambda _{n} = \lambda$, and G-MNL collapses to MXL. Further, there is no preference heterogeneity if $\eta _{n}$ = 0, thus, $\beta _{n} = \lambda _{n}\beta$. Two variants of G-MNL emerge if $\gamma$ is restricted to either zero (scaled random coefficients) or one (scaled means of the coefficients). In both cases, we will have $\beta _{n} = \lambda _{n}(\beta + \eta _{n})$ and $\beta _{n} = \lambda _{n}\beta + \eta _{n}$.

Another model that captures heterogeneity differently is the latent class logit model (LCM), because in modeling taste heterogeneity, corner solution may arise when a significant subpopulation of the population places a zero weight on some attributes and not accounting for this may not reveal the true nature of heterogeneity (Hensher, 2014). In modeling spatial heterogeneity, individuals are assumed to be sorted into a set of different classes or clusters (*c*), with the researcher not having prior knowledge of the cluster each belongs. Thus, preferences are homogeneous within classes but differ across classes (Greene & Hensher, 2003).

The difference between MXL and LCM is that in the former, parameters are individual-specific, while in the latter, it is class-specific. The utility is assigned a number based on the class to which a respondent belongs (Czajkowski et al., 2017). Further, while the MXL model assumes a full parametric distribution of the parameters, the LCM is semiparametric. This gives the analyst liberty not to make any distributional assumptions about individual heterogeneity (Greene & Hensher, 2003). While in the MXL, the coefficients are continuously distributed, they follow a discrete distribution in the LCM. Furthermore, although the two models can account for correlations between the coefficients, the analyst needs to specify this option in MXL while the LCM implicitly allows the coefficients to correlate. Thus, the choice of the distribution of $\beta$ in the LCM is not controversial. Finally, the MXL model is estimated through maximum simulated likelihood (SML), while the LCM is estimated via the ML approach (Hole, 2008).

The LCM probability that *n* makes a sequence of choices is specified as:

12 $$\begin{aligned} \begin{aligned} S_{n} =\sum ^{C}_{c=1}H_{nc}\prod ^{T}_{t=1}\prod ^{J}_{j=1}\left[ \frac{exp(\beta _{1c}stroke_{njt} +\cdots + \beta _{5c}payoff_{njt})}{\sum ^{J}_{j=1}exp(\beta _{1c}stroke_{njt} +\cdots + \beta _{5c}payoff_{njt})}\right] ^{y_{njt}} \end{aligned} \end{aligned}$$

where $H_{nc}$ is the probability that *n* belongs to class *c*, which gives the multinomial logit:

13 $$\begin{aligned} \begin{aligned} H_{nc} =\frac{exp(\gamma ^{\prime }_{c}Z_{n})}{\sum ^{C}_{=1}exp(\gamma ^{\prime }_{c}Z_{n})} \end{aligned} \end{aligned}$$

where $Z_{n}$ is a vector of observed characteristics of respondent *n* and $\gamma _{c}$ parameter is normalized to zero for model identification (Andersson et al., 2019; Greene & Hensher, 2003; Yoo, 2020).

The marginal WTA compensation for SHS exposure is derived by partially differentiating Eq. 7 with respect to each of the attributes and dividing each by the monetary attribute. As per the LCM, this is simulated using the class-specific marginal utilities.

14 $$\begin{aligned} \begin{aligned} mWTA= \frac{\partial U_{njt}/ \partial stroke_{njt}}{\partial U_{njt}/ \partial payoff_{njt}} = \left| \frac{\beta _{1}}{\beta _{5}}\right| \end{aligned} \end{aligned}$$

## Results

### Descriptive Statistics

Table 2 presents the key variables used in the analyses. We present the sample statistics of the pooled data, U.S., and U.K. data. For simplicity, we will focus on comparing U.S. and U.K. figures. In most cases, the samples are identical. The majority of the U.S. sample are males (55%) compared to 36%. More respondents are exposed to SHS at home and in private vehicles in the U.K. (35%) than in the U.S. (16%). Nearly half of respondents (48%) are living with a partner/spouse in the U.S. compared to 53% in the U.K. Only 4% of U.S. respondents’ partner/spouse smoke relative to 6% in the U.K. While 38% of U.S. respondents have/had serious ill-health, it is 40% in the U.K. The majority of respondents in the U.S. (82%) and U.K. (78%) indicated that SHS causes them distress. Respondents were asked to show on a ten-scale Likert of poor to excellent their knowledge about SHS effects. Most of the respondents (58%) in the U.S. and 53% of U.K. respondents reported having good knowledge, only 19% compared to 13% of respondents indicated that they have excellent knowledge. The income distributions of both samples are similar. The vast majority of respondents (64%) in the U.S. relative to 69% of U.K. respondents fall within the $1,100-$5,600 band.

Other variables not used in the estimations but provide more insight into our samples’ characteristics are presented in Table 14. The average age of U.S. respondents is 31.4 compared to 32.1 years. Only 24% (23%) of respondents have university degree; and 73% (69%) are in full employment; 63% (81%) are whites. It is worth noting that most U.K. respondents (72%) are exposed to SHS between four to seven times a week compared to 45% of U.S. participants. As per health-risk behaviors, more U.K. respondents (80%) consume alcohol compared to 56% of U.S. respondents. Further, they consume 3.4 glasses per week on average compared to 2.1 glasses reported by U.S. respondents.

**Table 2:** Summary statistics

Variable	Description	Mean (Std. Dev.)		
		Pooled	U.S.	U.K.
Male	= 1 if male	0.46 (0.499)	0.55 (0.498)	0.36 (0.482)
Exposure place	= 1 if exposed at home & vehicle	0.26 (0.437)	0.16 (0.369)	0.35 (0.478)
Living with partner/spouse	= 1 if living with partner	0.50 (0.500)	0.48 (0.500)	0.53 (0.499)
Smoking partner	= 1 if partner/spouse smokes	0.05 (0.219)	0.04 (0.193)	0.06 (0.243)
Health status	= 1 if suffered/suffering from serious ill-health	0.39 (0.488)	0.38 (0.485)	0.40 (0.490)
SHS distresses	= 1 if distressed by secondhand smoke	0.80 (0.400)	0.82 (0.384)	0.78 (0.417)
KSHS: good	= 1 if respondent has good knowledge about SHS risks	0.56 (0.497)	0.58 (0.494)	0.53 (0.499)
KSHS: excellent	= 1 if respondent has Excellent knowledge	0.16 (0.368)	0.19 (0.395)	0.13 (0.336)
*Income: $1100–$5,600	= 1 if yes	0.66 (0.473)	0.64 (0.481)	0.69 (0.463)
Income: $5601–$10,100	= 1 if yes	0.08 (0.273)	0.15 (0.359)	0.01 (0.105)
Income: More than $10,100	= 1 if yes	0.04 (0.205)	0.07 (0.258)	0.02 (0.128)

*Monthly disposable income

#### Perceptions About Smoking Control

For exploratory reasons, we elicited respondents’ attitudes towards smoking. First, we asked: “Should governments ban smoking at home and in private vehicles when a nonsmoker is present?” An equal proportion of U.S. respondents (37%) voted yes and no, respectively. At the same time, 26% did not care. In the U.K., 60% of respondents answered in affirmative, only 16% voted no, and 24% did not care. Finally, we asked: “Should governments empower kids exposed to secondhand smoke at home to sue the smoker when they become adults?” Again, there were cross-country discrepancies. Only 23% of U.S. respondents answered yes, 32% indicated no, and 45% did not care. In comparison, 33% of U.K. respondents supported the law, 27% were against it, and the majority 40% did not care. These discrepancies could be because more U.K. respondents are exposed to SHS in those places than U.S. participants.

It is important to note that the statistical information presented herein pertains to a subset of the sample, as not all respondents responded to the open-ended questions. Consequently, to mitigate the potential for a substantial amount of missing data, which could compromise the robustness of the outcomes, we opted to avoid incorporating perceptions as an independent predictor in the models. Nonetheless, the descriptive analysis yields valuable insights into nonsmokers’ perceptions regarding smoking control in both countries.

### CV Results

Diagnostic tests reveal that the distribution of WTP skewed to the left, and using it at levels can lead to biased predictions because it forces the effects of the independent variables to be additive (Cameron & Trivedi, 2010). Thus, we conduct the Box-Cox specification test on the log of WTP. The test favors the log-linear specification. We further test the functional form of the conditional mean of ln(WTP) using the Ramsey RESET test. The results show that the conditional mean of ln(WTP) is correctly specified. Although the standard errors are specified as robust, we formally test for the presence of heteroskedasticity using the Breusch-Pagan/Cook-Weisberg test. We conduct robustness checks using quantile regression (median regression).

We pooled the U.S. and U.K. data and estimate the difference using a dummy variable and further estimate separate models using country-specific data. The coefficient of the dummy variable in Table 3 is positive and statistically significant. This shows that U.S. respondents value SHS health risks more than their U.K. counterparts. In both countries, WTP declines with female respondents. Suspecting that the income effects might be responsible for this, we interact income with gender, but it is consistently negative and insignificant. The results are presented in Table 15. U.S. respondents who are living with a partner or spouse value SHS health risks less. However, those whose partner/spouse smokes in both countries are willing to pay more to prevent further SHS exposure. U.K. respondents suffering from or have experienced a serious ill-health are willing to pay more to avert SHS exposure. Also, U.K. respondents exposed to SHS at home/vehicle and are distressed by it value it more than their counterparts. In terms of knowledge, only U.S. respondents with excellent knowledge about SHS health effects are willing to pay more to prevent exposure. Furthermore, income is a significant predictor of WTP.

**Table 3:** OLS and tobit models estimates (dep. var: ln(WTP))

Variable	Pooled		U.S.		U.K.	
	OLS	Tobit	OLS	Tobit	OLS	Tobit
Dummy$$^{\dagger }$$	0.503*** (0.136)	0.499*** (0.133)				
Male	0.622*** (0.122)	0.615*** (0.123)	0.412** (0.180)	0.402** (0.183)	0.832*** (0.163)	0.827*** (0.163)
Living with partner	− 0.342*** (0.130)	− 0.347*** (0.130)	− 0.538*** (0.194)	− 0.558*** (0.198)	− 0.166 (0.180)	− 0.157 (0.167)
Partner smokes	1.099*** (0.259)	1.104*** (0.286)	1.123** (0.556)	1.146** (0.507)	1.169*** (0.249)	1.162*** (0.332)
Health status	0.073 (0.125)	0.076 (0.124)	− 0.238 (0.192)	− 0.222 (0.189)	0.374** (0.160)	0.366** (0.158)
Exposure place$$\times$$distress	0.573** (0.232)	0.577** (0.222)	0.397 (0.397)	0.495 (0.361)	0.791*** (0.301)	0.792*** (0.277)
SHS knowledge: good	0.056 (0.141)	0.045 (0.142)	0.360 (0.224)	0.345 (0.228)	− 0.190 (0.179)	− 0.203 (0.174)
SHS knowledge: excellent	0.106 (0.203)	0.086 (0.195)	0.573* (0.305)	0.552* (0.290)	− 0.289 (0.251)	− 0.304 (0.261)
Income: $1100–$5600	0.232 (0.152)	0.224 (0.157)	0.379 (0.259)	0.344 (0.275)	0.054 (0.186)	0.061 (0.182)
Income: $5601–$10100	0.503* (0.296)	0.481* (0.269)	0.665* (0.353)	0.634* (0.352)	1.422 (0.896)	1.427* (0.753)
Income: more than $10100	0.873** (0.338)	0.869*** (0.329)	1.246*** (0.408)	1.226*** (0.432)	− 0.117 (0.559)	− 0.109 (0.633)
Constant	3.333*** (0.210)	3.349*** (0.223)	3.893*** (0.334)	3.928*** (0.301)	3.196*** (0.265)	3.201*** (0.268)
σ		2.614 (0.138)		2.937 (0.221)		2.143 (0.159)
Log-likelihood		− 1378.003		− 709.256		− 653.394
R-squared	0.122	0.032	0.093	0.023	0.144	0.042
Obs.	727	727	364	364	363	363

***$p<$0.01, **$p<$0.05, *$p<$0.10. Standard errors (in parentheses) are robust. $\dagger$ =1 if U.S. and 0 if U.K. The tobit model is left and right-censored

Table 4 presents the results of the quantile or median regression, using the 50th and 75th percentiles. It is worthy of note here that the gender discrepancy still holds in both countries. Thus, it is sufficient to conclude that female respondents value SHS risks less than men. In most cases, the signs of the coefficients are not different from those in Table 3.

**Table 4:** Quantile regression model estimates

Variable	U.S.		U.K.	
	Q (0.50)	Q (0.75)	Q (0.50)	Q (0.75)
Gender	0.536*** (0.151)	0.523** (0.247)	0.733*** (0.204)	0.733*** (0.129)
Living with partner	− 0.313* (0.169)	− 0.523** (0.249)	− 0.182 (0.225)	–
Partner smokes	0.562 (0.871)	1.347*** (0.294)	1.057*** (0.291)	0.693*** (0.224)
Health status	− 0.156 (0.151)	− 0.562** (0.226)	0.510** (0.205)	0.111 (0.132)
Exposure place$$\times$$distress	0.941*** (0.320)	0.562 (0.478)	0.952*** (0.337)	0.763** (0.325)
SHS knowledge: good	0.156 (0.171)	0.392 (0.254)	− 0.405* (0.233)	− 0.223 (0.222)
SHS knowledge: excellent	0.156 (0.226)	0.036 (0.324)	–	− 0.293 (0.208)
Income: $1100–$5600	0.536*** (0.189)	0.379 (0.501)	0.146 (0.211)	0.182 (0.223)
Income: $5601–$10,100	0.693** (0.300)	0.680 (0.632)	1.427* (1.427)	1.457 (2.041)
Income: more than $10,100	1.22*** (0.452)	1.427*** (0.529)	0.551 (0.979)	0.111 (0.911)
Constant	3.532*** (0.224)	4.748*** (0.545)	3.022*** (0.325)	4.226*** (0.325)
R-squared	0.031	0.069	0.098	0.062
Obs.	364	364	363	363

***$p<$0.01, **$p<$0.05, *$p<$0.10. Standard errors (in parentheses) are robust

We present the mean and median WTP of respondents in Table 5. U.S. respondents are willing to pay $521.63 to prevent SHS exposure compared to $242.96 by U.K. respondents. The difference is statistically significant ($$p<0.01$$). The median WTP is $100 compared to $68.57. We extrapolate these figures to the population to derive the economic cost of secondhand smoke. The costs are $173.123 billion and $16.357 billion in the U.S. and U.K., respectively.

**Table 5:** WTP (U.S. & U.K.)

	Mean	Median	Obs.
U.S.	$521.63 (1861.92)	$100.00	364
U.K	$242.96 (1516.98)	$68.57	363

Standard deviations are in parentheses

### CE Results

To ensure that respondents did not engage in unethical practices, we checked the possibility of consistently choosing a particular option before the estimation (see Viscusi et al., 1991). We did not find any abnormal responses, but incomplete responses were deleted. Following best practice guidelines (see Johnston et al., 2017, Lancsar et al., 2017), we started with a simple model such as the conditional logit (or fixed effect model) in Eq. 8 and estimated more advanced ones like the mixed logit/random parameters logit specified in Eq. 9, and generalized multinomial logit (G-MNL) in Eq. 11. While the conditional logit model treats individual preferences as homogeneous, the more advanced models account for heterogeneity in taste and scale. We model all the variables as continuous, except the emotional distress variable coded as a dummy. See Tables 12 and 13 for the summary statistics of the choice models variables.

In MXL I, we treat all parameters as normally distributed, except the payoff parameter, whereas all parameters are normally distributed in MXL II. MXL III accounts for correlation among the random coefficients. Since each respondent completed eight tasks, we anticipate correlated responses. Failure to account for this could bias the results (see Carlsson et al., 2010).

In the G-MNL specification, we restrict gamma to zero to account for scale heterogeneity (differences in error variance). Failure to account for this could produce biased estimators (Fiebig et al., 2010; Haab et al., 1999; Johnston et al., 2017). This model collapses to MXL if the coefficient of the scale parameter turns out to be statistically insignificant. The MXL and G-MNL models were estimated through maximum simulated likelihood with 500 Halton draws. We circumvent confounding effects on our results by not including covariates.

Concerning the coefficients’ interpretation of the results in Tables 6 and 7, it should be noted that signs of the attributes’ coefficients relate to how each affects the dependent variable (choice probability). A negative coefficient shows the probability of a decrease in utility. Overall, the signs of the coefficients are consistent with a priori expectation. All the coefficients are statistically significant. U.S. respondents prefer policies with a higher reduction in health insurance premiums and lower SHS health risks. However, the coefficient of a tax reduction in Table 7 is not statistically different from zero. It is worthy to note that respondents prefer a higher probability of reducing the risk of emotional distress than other risks.

It can be seen that the standard deviation coefficients are significant, meaning that there is evidence of heterogeneity in taste across our samples. Further, the scale parameter in the G-MNL is significant, indicating scale heterogeneity. In Table 6, the AIC and BIC favor MXL II, where all the coefficients are treated as random. However, the outcome is mixed in Table 7, where the AIC favors MXL III, while BIC prefers MXL II. The log-likelihood function is higher in MXL III. Revelt and Train (1998) recommended modeling the monetary variable as fixed; however, like our results, Meijer and Rouwendal (2006) and Hole (2008) found that allowing it to vary fits their data better.

**Table 6:** Estimates of choice models (U.S.)

Variable	CL	MXL I	MXL II	MXL III	G-MNL
	Coeff. (S.E.)	Coeff. (S.E.)	Coeff. (S.E.)	Coeff. (S.E.)	Coeff. (S.E.)
Stroke risk	− 0.051*** (0.004)	− 0.078*** (0.008)	− 0.093*** (0.009)	− 0.082*** (0.010)	− 0.114*** (0.019)
Lung cancer risk	− 0.065*** (0.003)	− 0.111*** (0.007)	− 0.126*** (0.009)	− 0.118*** (0.007)	− 0.194*** (0.042)
Coronary heart disease risk	− 0.042*** (0.002)	− 0.072*** (0.005)	− 0.081*** (0.006)	− 0.084*** (0.007)	− 0.122*** (0.024)
Emotional distress risk	− 0.583*** (0.049)	− 0.810*** (0.081)	− 0.885*** (0.094)	− 0.754*** (0.099)	− 1.414*** (0.321)
Health insurance premium	0.008*** (0.002)	0.015*** (0.003)	0.018*** (0.004)	0.013*** (0.004)	0.017*** (0.006)
*Standard deviation*					
Stroke risk		0.060*** (0.018)	0.051* (0.027)	0.084*** (0.018)	0.047 (0.050)
Lung cancer risk		0.084*** (0.006)	0.098*** (0.007)	0.086*** (0.006)	0.133*** (0.028)
Coronary heart disease risk		0.061*** (0.006)	0.071*** (0.006)	0.072*** (0.008)	0.090*** (0.017)
Emotional distress risk		0.928*** (0.104)	0.982*** (0.118)	0.948*** (0.113)	1.218*** (0.287)
Health insurance premium				0.054*** (0.007)	
$\tau$					− 1.051*** (0.193)
Observations	8656	8656	8656	8656	8656
Number of respondents	541	541	541	541	541
LL	− 2052.3809	− 1892.622	− 1870.189	− 1867.287	− 1887.0305
AIC	4114.762	3803.244	3760.378	3764.574	3794.061
BIC	4150.092	3866.838	3831.038	3870.564	3864.721

***$p<$0.01, **$p<$0.05, *$p<$0.10. Standard errors (in parentheses) are robust. *CL* conditional logit, *MXL* mixed logit, *G-MNL* generalized multinomial logit. MXL and G-MNL models were estimated with Stata 16

**Table 7:** Estimates of choice models (U.K.)

Variable	CL	MXL I	MXL II	MXL III	G-MNL
	Coeff. (S.E.)	Coeff. (S.E.)	Coeff. (S.E.)	Coeff. (S.E.)	Coeff. (S.E.)
Stroke risk	− 0.049*** (0.004)	− 0.072*** (0.006)	− 0.083*** (0.007)	− 0.093*** (0.008)	− 0.169*** (0.039)
Lung cancer risk	− 0.071*** (0.003)	− 0.121*** (0.007)	− 0.134*** (0.009)	− 0.128*** (0.008)	− 0.258*** (0.064)
Coronary heart disease risk	− 0.044*** (0.002)	− 0.075*** (0.005)	− 0.083*** (0.005)	− 0.083*** (0.007)	− 0.154*** (0.042)
Emotional distress risk	− 0.670*** (0.050)	− 0.905*** (0.079)	− 0.984*** (0.089)	− 0.9034*** (0.099)	$-$1.978*** (0.452)
Tax	0.0004 (0.002)	0.003 (0.004)	0.005 (0.004)	0.001 (0.004)	− 0.004 (0.011)
*Standard deviation*					
Stroke risk		− 0.037*** (0.007)	0.010 (0.014)	0.079*** (0.012)	− 0.037 (0.0312)
Lung cancer risk		0.095*** (0.007)	0.109*** (0.008)	0.099*** (0.007)	0.176*** (0.032)
Coronary heart disease risk		0.051*** (0.005)	0.063*** (0.008)	0.059*** (0.008)	0.039*** (0.014)
Emotional distress risk		0.817*** (0.099)	0.749*** (0.126)	0.846*** (0.094)	1.546*** (0.602)
Tax				0.056*** (0.006)	
$\tau$					− 1.240*** (0.209)
Observations	8864	8864	8864	8864	8864
Number of respondents	554	554	554	554	554
LL	− 2049.3869	− 1853.223	− 1829.104	− 1816.832	− 1835.985
AIC	4108.774	3724.446	3678.209	3663.664	3691.969
BIC	4144.223	3788.253	3749.106	3770.01	3762.867

***$p<$0.01, **$p<$0.05, *$p<$0.10. Standard errors (in parentheses) are robust. *CL* conditional logit, *MXL* mixed logit, *G-MNL* generalized multinomial logit. MXL and G-MNL models were estimated with Stata 16

### Latent Class Model Results

Since the MXL model results show heterogeneity in our samples, we use a latent class logit model to sort respondents into different groups comprising identical preferences. We estimate three classes, which are determined using AIC and BIC. It is assumed that preferences are homogeneous within a class but heterogeneous across classes (Greene and Hensher, 2003). Ultimately, the model allows us to see how respondents in each class value the attributes. In our latent class model, class membership is explained by a constant. Thus, the likelihood of belonging to each group is constant across respondents (see also Adamowicz et al., 2011, Hole, 2008).

We model the payoff as homogeneous in Table 8, while we allow it to vary in Table 9. Specifying it this way gives our data a better fit. All the coefficients in Table 8, have the expected signs and are statistically significant, except the coefficient of emotional distress in class 3. This shows that emotional risk is not paramount to about 30% of respondents. Indeed, SHS may not cause emotional distress to a nonsmoker, depending on where their exposure takes place and the frequency. Furthermore, the log-likelihood is similar to the mixed logit model in Table 8, where all the coefficients are allowed to vary. This is consistent with the findings of Hole (2008). The majority of respondents belong to class 1, followed by classes 3 and 2, respectively.

As per the U.K. results, Table 9 shows that all the coefficients have the expected signs, except in class 2, where the tax coefficient is negative. It can be seen that the results vary substantially across classes. Most of the coefficients in class 2 are statistically significant, while only coronary heart disease risk and emotional distress are significant in class 1. In class 3, stroke and lung cancer risks are significant. The vast majority of respondents belong to class 1, followed by classes 2 and 3, respectively. Although the results of CL, MXL, and G-MNL presented in Table 7 show that the tax coefficient is not significant, the latent class model helps us see that it is significant for class 3 respondents.

**Table 8:** Latent class logit model estimates (U.S.)

Variable	Class 1	Class 2	Class 3
	Coeff. (S.E.)	Coeff. (S.E.)	Coeff. (S.E.)
Stroke risk	− 0.117*** (0.014)	− 0.035*** (0.009)	− 0.047*** (0.018)
Lung cancer risk	− 0.088*** (0.010)	− 0.012*** (0.005)	− 0.339** (0.174)
Coronary heart disease risk	− 0.093*** (0.009)	− 0.005* (0.005)	− 0.051*** (0.0188)
Emotional distress risk	− 1.106*** (0.172)	− 0.240*** (0.083)	− 2.020 (1.704)
Health insurance premium	0.011*** (0.003)		
Constant	0.362* (0.210)	− 0.097 (0.189)	
Class share	0.429	0.272	0.299
Observations	8656		
Number of respondents	541		
LL	− 1870.319		

***$p<$0.01, **$p<$0.05, *$p<$0.10. Standard errors are in parentheses. Health insurance premium is specified as homogeneous

**Table 9:** Latent class logit model estimates (U.K.)

Variable	Class 1	Class 2	Class 3
	Coeff. (S.E.)	Coeff. (S.E.)	Coeff. (S.E.)
Stroke risk	− 0.013 (0.010)	− 0.135*** (0.018)	− 0.079*** (0.022)
Lung cancer risk	− 0.001 (0.006)	− 0.126*** (0.017)	− 0.296*** (0.040)
Coronary heart disease risk	− 0.019*** (0.004)	− 0.098*** (0.012)	− 0.032 (0.026)
Emotional distress risk	− 0.377*** (0.110)	− 1.757*** (0.381)	− 0.778 (0.479)
Tax	0.009 (0.008)	− 0.037*** (0.015)	0.048*** (0.016)
Constant	− 0.245 (0.188)	0.432** (0.199)	
Class share	0.480	0.293	0.228
Observations	8864		
Number of respondents	554		
LL	− 1803.912		

***$p<$0.01, **$p<$0.05, *$p<$0.10. Standard errors are in parentheses

Table 10 presents the estimated WTA and 95% confidence intervals. The WTA estimates derived from the MXL II model are $3,067.57 for a potential stroke risk, $4,138.16 for lung cancer risk, $2,677.89 for coronary heart disease risk, and $28,939.14 for emotional distress risk. Comparing the estimates of the three classes in the latent class model, the WTA are $6,410.28, $1,912.11, and $2,606.68 for stroke risk; $4,809.25, $668.09, and $18,606.71 for lung cancer risk; $5,114.62, $515.28, and $2,822.96 for coronary heart disease; $60,766.10 and $13,210.01 for emotional distress, respectively.

Regarding the U.K. results presented in Table 11, we estimate the WTA of class 3 only because the tax coefficient is either not in line with theory or not statistically significant in other classes. The WTA is $3,424.69 for stroke risk and $12,731.80 for lung cancer risk.

**Table 10:** WTA (U.S.)

Variable	CL	G-MNL	MXL I	MXL II	MXL III	Class 1	Class 2	Class 3
	Coeff. (C.I)	Coeff. (C.I)	Coeff. (C.I)	Coeff. (C.I)	Coeff. (C.I)	Coeff. (C.I)	Coeff. (C.I)	Coeff. (C.I)
Stroke risk	3496.92 (1393.98–5599.85)	3806.13 (1117.19–6495.08)	2944.55 (1479.60–4409.50)	3067.57 (1591.83–4543.32)	3661.52 (1395.70–5927.35)	6410.28 (2809.77–10010.79)	1912.11 (465.26–3358.96)	2606.68 (220.09–4993.27)
Lung cancer risk	4399.07 (1750.21–7047.92)	6457.12 (1636.47–11277.75)	4184.77 (2174.05–6195.50)	4138.16 (2161.25–6115.07)	5277.55 (2057.80–8497.30)	4809.25 (1972.04–7646.46)	668.09 (35.82–1300.36)	18606.71 (2498.99–39712.41)
Coronary heart disease risk	2842.71 (1136.42–4548.99)	4065.88 (931.67–7200.09)	2706.06 (1393.83–4018.28)	2677.89 (1400.47–3955.31)	3783.85 (1461.05–6106.65)	5114.62 (2280.57–7948.67)	515.28 (77.26–1107.83)	2822.96 (39.41–5685.33)
Emotional distress risk	39271.49 (13687.92–64855.05)	46936.86 (10947.33–82926.40)	30419.28 (14221.10–46617.46)	28939.14 (13632.80–44245.47)	33645.82 (10159.78–57131.85)	60766.10 (18568.02–102964.20)	13210.01 (2247.68–24172.33)	DNA

95% confidence interval in parentheses was simulated through the Delta method. DNA means does not exist because the estimate is not statistically significant. The coefficients, which were derived by multiplying the WTA formula by the respondents’ average monthly health insurance premiums, are in US$

**Table 11:** Latent class: WTA (U.K.)

Variable	Class 1	Class 2	Class 3
	Coeff. (C.I)	Coeff. (C.I)	Coeff. (C.I)
Stroke risk	DNA	DNA	3424.69 (942.974–5906.40)
Lung cancer risk	DNA	DNA	12731.8 (6536.89–18926.7)
Coronary heart disease risk	DNA	DNA	DNA
Emotional distress risk	DNA	DNA	DNA

95% confidence interval in parentheses was simulated through the Delta method. DNA means does not exist. The coefficients, which were derived by multiplying the WTA formula by the respondents’ average monthly tax are in USD at £/1.37USD

## Discussion and Conclusion

Several noteworthy insights emerge from our study findings. Regarding the contingent valuation (CV) results, as presented in Table 3, it is evident that American respondents exhibit a higher WTP  than their British counterparts. The underlying determinants for this divergence are multifaceted. One plausible explanation is rooted in the premise that American respondents may possess a higher degree of health risk aversion relative to their British counterparts. This observation aligns with established research indicating that individuals with heightened risk aversion tendencies tend to manifest a greater WTP in order to mitigate health risks (Congress, 1997; Eeckhoudt & Hammitt, 2004; Fuchs & Zeckhauser, 1987; Liu et al., 1997; Smith et al., 2004).

These outcomes carry substantial implications for public policy formulation and regulatory measures. Recognizing the variances in risk aversion between American and British nonsmokers prompts a nuanced approach to shaping smoke-free policies in both countries. In the United States, policy interventions that effectively communicate the tangible health risks of secondhand smoke exposure may resonate more deeply with the population, particularly those inclined towards risk aversion. Enhancing awareness campaigns and disseminating information regarding the heightened health risks of secondhand smoke could garner more public support for stringent smoking control measures.

Conversely, while the general WTP appears lower in the British context, policy strategies could be tailored to resonate with the prevailing societal norms and preferences. Since policy preferences often reflect broader socio-cultural contexts, initiatives emphasizing the collective benefits of reduced secondhand smoke exposure within the British populace may resonate more strongly. Acknowledging these contrasting psychological drivers can inform the design and implementation of culturally relevant anti-smoking campaigns and public health interventions.

Our findings not only underscore the disparities in WTP for secondhand smoke health risks between American and British nonsmokers but also underscore the role of risk aversion as a potential underlying factor. These insights substantiate the significance of tailored policy approaches grounded in the nuanced psychological predispositions of the respective populations, thus facilitating the development of more effective and resonant smoke-free policies in both countries.

Our research findings indicate that male respondents are willing to allocate a higher monetary value in comparison to their female counterparts for mitigating the health risks associated with secondhand smoke. A plausible explanation for this disparity might be that male nonsmokers demonstrate a higher degree of health risk aversion compared to female nonsmokers. Nevertheless, it is important to note that the literature on valuation of health risks presents a mixed array of results. Some studies align with our observations (Adamowicz et al., 2011; Frew et al., 2001; Neumann et al., 2012; Tubeuf et al., 2015), while others contradict these outcomes (Andersson et al., 2015; Condliffe & Fiorentino, 2014; Viscusi & Huber, 2012).

Initially, we considered the possibility that other underlying factors, such as income level, might account for these divergent findings. However, after incorporating interaction terms in the analytical model (as presented in Table 15), we found no significant evidence to support such a relationship. The coefficients associated with the interaction terms were statistically insignificant, albeit displaying counterintuitive signs.

This insight into gender-based variations in the valuation of secondhand smoke health risks holds significant implications for public policy and regulatory decisions in both the United States and the United Kingdom. Understanding the differential willingness to pay for mitigating secondhand smoke health risks between male and female nonsmokers can aid policymakers in designing more targeted and effective interventions.

For instance, if male nonsmokers are indeed more risk-averse and willing to allocate higher financial resources for reducing their exposure to secondhand smoke, policymakers might consider tailoring educational campaigns and health initiatives specifically towards this demographic. Moreover, it could warrant a closer examination of the factors influencing risk perception and aversion in each gender group to address potential gender-specific barriers and concerns.

Additionally, by recognizing the mixed findings in the existing valuation literature, policymakers must approach this matter with caution and ensure that regulations and policies are well-informed by robust scientific evidence. Continued research and validation of these findings through longitudinal studies could provide a more comprehensive understanding of the underlying mechanisms influencing gender disparities in risk perception and willingness to pay for health protections.

In summary, this results shed light on the differential valuation of secondhand smoke health risks between male and female nonsmokers. The understanding of such gender-based disparities can contribute to the formulation of more targeted and effective public health policies, leading to better protection for both nonsmokers and the general population from the adverse effects of secondhand smoke exposure in the U.S. and U.K.

Notably, respondents with a smoker partner or spouse demonstrate a higher WTP, consistent with existing literature. Noteworthy studies have demonstrated that nonsmokers living with a partner or spouse who smokes face a substantially 30% higher risk of developing lung cancer than their counterparts residing with non-smoking partners or spouses (Hirayama, 1984; Pressman, 1993). Although complex, this association underscores the direct impact of exposure to secondhand smoke within intimate settings on perceived health risks and, subsequently, the corresponding valuation of strategies to reduce such risks.

Furthermore, our findings underscore a distinct trend within the British context. British respondents who have encountered episodes of significant ill health in their lives exhibit an increased willingness to pay  to mitigate secondhand smoke risks. This inclination can be attributed to a heightened awareness of health vulnerabilities and a consequential desire to avoid exacerbating existing health conditions. Similar observations have been reported in prior research, supporting the notion that individuals who have experienced health setbacks are more attuned to the potentially detrimental effects of environmental factors such as SHS (Andersson et al., 2015; Frew et al., 2001; Hammitt & Zhou, 2006).

These discernible trends within our results carry pivotal implications for policy formulation and public health interventions. The elevated WTP among respondents cohabiting with smoker partners or spouses emphasizes the imperative for tailored campaigns and strategies that address not only the health risks of the smokers themselves but also those imposed on their non-smoking counterparts within shared living environments. Awareness initiatives targeting smokers and their cohabitants, emphasizing the dangers of secondhand smoke, may drive behavior change and incentivize more stringent smoke-free household policies.

Moreover, recognizing the augmented valuation of SHS risk mitigation strategies among British respondents who have faced health adversities underscores the potential effectiveness of targeted health communication campaigns. Designing public health messages that resonate with individuals who have experienced ill health can reinforce the importance of minimizing environmental health risks, fostering a greater willingness to support measures to reduce SHS exposure financially.

Our study’s identification of these distinct trends in WTP among respondents with smoker partners or spouses and those with previous experiences of ill health provides valuable insights for shaping policies that more effectively address secondhand smoke health risks. Tailored interventions, taking into account these nuanced factors, hold the potential to instigate behavioral shifts and promote healthier living environments, thereby contributing to enhanced public health outcomes.

Individuals exposed to SHS within their residences and private vehicles exhibit an elevated WTP to evade such exposure. However, it is notable that the statistical significance of this result is not observed within the U.S. context. This lack of significance could be attributed to the disparity in exposure prevalence, with 35% of U.K. respondents experiencing SHS exposure in these environments, in contrast to 16% of their U.S. counterparts. This variance in exposure rates underscores the intricate interplay between personal exposure experiences and the valuation of risk mitigation strategies.

Moreover, within the U.S. context, respondents demonstrating a heightened awareness of the health effects of SHS exhibit a higher WTP. This trend resonates with the findings of Kenkel (1991), who observed a decrease in health-risk behaviors among Americans possessing comprehensive health knowledge. This alignment highlights the pivotal role of knowledge dissemination in influencing individuals’ risk perceptions and willingness to allocate resources for health risk abatement.

The influence of socioeconomic status on WTP is also notable. Wealthier respondents showcase a higher WTP, a pattern congruent with existing research (Andersson et al., 2015, 2019; Adamowicz et al., 2011; Eeckhoudt & Hammitt, 2004; Viscusi & Huber, 2012). This phenomenon can be attributed to the recognition that individuals with greater economic resources possess more to lose in the event of health afflictions, thus motivating a more pronounced willingness to invest in averting potential health risks.

The substantial discrepancy between U.S. and U.K. respondents’ mean WTP is a striking outcome. U.S. respondents exhibit a WTP that is double that of their U.K. counterparts. The magnitude of this discrepancy is accentuated when contextualized against previous studies. U.S. nonsmokers’ mean WTP surpasses the $494 that American smokers were willing to pay annually to shield their children from SHS exposure (Agee et al., 2001). Additionally, U.S. respondents’ WTP surpasses the respective CAN$100 and CAN$225 values Canadians were willing to pay to reduce respiratory and cardiovascular diseases (Johnson et al., 2000). When compared internationally, Chinese respondents demonstrated a notably higher valuation of risks associated with chronic bronchitis and asthma due to air pollution (Guo et al., 2006; Hammitt & Zhou, 2006; Peng & Tian, 2003).

In this scenario, where current partial smoke-free laws persist, our analysis reveals significant disparities in the projected costs of SHS between the United States and the United Kingdom. Specifically, the estimated cost of SHS exposure is calculated to amount to $173.123 billion in the U.S., in stark contrast to $16.357 billion in the U.K.

These substantial variations in estimated costs can be attributed to several underlying factors. Firstly, the dissimilarity in population size between the two countries inevitably contributes to the stark contrast in absolute cost estimates. The larger population in the U.S. and the higher smoking prevalence rates result in an escalated burden of SHS-related health risks and their attendant costs. Moreover, disparities in SHS exposure prevalence and intensity within different societal contexts are pivotal contributors. In the U.S., despite partial smoke-free laws, a notable prevalence of SHS exposure persists due to localized variations in regulatory stringency and compliance. Conversely, the U.K.’s more comprehensive smoke-free legislation has significantly reduced smoking, leading to correspondingly lower projected costs.

These findings possess profound implications for policy formulation and public health interventions. The stark contrast in projected SHS costs between the U.S. and the U.K. underscores the potency of stringent smoke-free legislation in effectuating meaningful reductions in smoking-related health burdens. Robust smoke-free laws in the U.K. have successfully curtailed SHS exposure, yielding substantial health and economic dividends (WHO, 2023). This phenomenon underscores the imperative for the U.S. to consider augmenting its existing regulatory framework to encompass more comprehensive smoke-free policies, thereby mitigating the projected economic burden of SHS exposure.

Furthermore, our study underscores the latent economic value of bolstered anti-smoking policies. The pronounced cost disparities underscore the potential for substantial savings that can be realized by adopting measures to reduce SHS exposure. By integrating these economic insights into policy dialogues, public health advocates and policymakers can articulate the fiscal merits of comprehensive smoke-free legislation, fostering political will and facilitating informed decision-making.

In sum, the estimated economic costs of SHS exposure within a business-as-usual context are a stark reminder of insufficient smoke-free regulations’ tangible health and economic ramifications. The disparities in cost projections between the U.S. and the U.K. underscore the pivotal role of stringent regulatory measures in ameliorating SHS-related health burdens. These findings advocate for prioritizing robust smoke-free legislation, offering substantial economic benefits while safeguarding public health on both sides of the Atlantic.

The examination of the choice experiment results, as delineated in Table 7, elucidates a discernible contrast in the responses of U.K. and U.S. respondents. Specifically, U.K. respondents exhibit a stance of indifference towards a prospective policy entailing a guaranteed augmentation in disposable income as compensation for exposure to secondhand smoke (SHS). Conversely, U.S. respondents manifest an opposing inclination, displaying a preference for such compensation. This dichotomy of response can be explicated through the prism of several intricate factors, including variances in healthcare systems, as well as considerations of altruism, warm glow, and social preferences.

The observed distinctions in response may partly be attributed to the divergent healthcare systems in place. The U.S. healthcare framework, characterized by a greater emphasis on individualism and private provision, could foster a heightened valuing of individual compensation. Conversely, the publicly funded healthcare system in the U.K. may cultivate a sense of collectiveness and community well-being, thereby attenuating the perceived necessity for individual financial recompense. This perspective resonates with the observations of Bridges (2003), who linked social preferences to the concept of social capital-a construct encompassing shared communal values in contrast to individualistic paradigms.

Additionally, the propensity of U.K. respondents to favor mitigation measures over compensation merits consideration. This phenomenon aligns with the findings of Knetsch (1990), who established a preference for direct action to alleviate environmental concerns rather than material compensation. Furthermore, insights from Hollinghurst et al. (2016) highlight the intricacy of risk perception within the U.K. context. As indicated by Hollinghurst and colleagues, the inconsistency in participant responses underscores the nuanced and multifaceted nature of how U.K. individuals appraise and engage with risk-related scenarios.

These nuanced findings offer pertinent implications for policy formulation and strategic interventions. The preference divergence between U.K. and U.S. respondents underscores the imperative of contextually tailored policy approaches. For the U.S., where compensation is favored, policy strategies that offer material incentives could align with the prevailing individualistic ethos. In contrast, in the U.K., where mitigation is seemingly valued, policies prioritizing comprehensive smoke-free initiatives and health promotion measures may find greater resonance. Recognizing the interconnectedness between healthcare frameworks, societal norms, and valuation mechanisms is critical in crafting strategies that effectively cater to distinct preferences.

The juxtaposition of U.K. and U.S. respondent responses underscores the intricate interplay of healthcare systems, cultural inclinations, and valuation paradigms. These findings impel policymakers to adopt strategies that resonate with the prevailing societal ethos, facilitating more effective policy implementation. By aligning interventions with the nuanced preferences of each context, opportunities for fostering healthier environments and mitigating secondhand smoke-related health risks can be optimally harnessed.

The analysis of the findings, as elucidated in Table 9, engenders a deeper comprehension of the respondent preferences. This stratification of individuals into three distinct segments offers valuable insights into the multifaceted landscape of preferences exhibited by the study participants. The outcomes notably highlight the emergence of discrete segments characterized by unique valuation patterns, delineating three specific respondent groups.

Firstly, our analysis discerns a segment within the U.K. nonsmoking population that attributes a tangible monetary value to the health risks associated with stroke and lung cancer. This notable segment may encompass individuals who possess private health insurance or lack registration with a General Practice (GP), thus relying on direct out-of-pocket expenditures to access healthcare services. Alternatively, it is plausible that a significant proportion of respondents exposed to SHS within private settings align with this segment.

Parallel to our findings, extant literature has reported analogous class distinctions in respondents’ valuations of cancer disease attributes (Adamowicz et al., 2011; Andersson et al., 2016). These collective findings underscore the consistency of such segmental differentiation across different study contexts and settings, thus emphasizing the generalizability of the observed phenomenon. Informed by these consistent trends, policymakers can derive insights for formulating targeted communication strategies and policy frameworks that resonate with the distinct valuation patterns exhibited by these segments.

Examining emotional distress, an aspect hitherto underrepresented within the health valuation literature, introduces a fresh and noteworthy dimension to our study. Evidentiary support for emotional distress among Americans exposed to SHS has been documented (Bandiera et al., 2011), thereby elevating the pertinence of its inclusion in the valuation framework.

The outcomes articulated in Table 10 illuminate a distinctive tendency among American respondents to attribute a higher monetary value to emotional distress when compared to other associated attributes. The rationale underpinning this pronounced valuation preference warrants exploration. The precise factors engendering this elevated valuation for emotional distress in contrast to other attributes remain unresolved. However, insights from the American Psychiatric Association (APA) provide valuable context.

Indeed, the American Psychiatric Association’s assertion that mental health issues manifest as a substantial societal concern within the U.S., impacting nearly one in five adults (APA, 2018), offers a vantage point for comprehending the observed valuation patterns. The omnipresence of mental health struggles underscores the palpable influence of emotional well-being on the broader fabric of American society. The potential link between SHS exposure and subsequent emotional distress within a nation grappling with a significant mental health landscape signifies a salient policy consideration.

Policy insights stemming from these findings advocate for multifaceted interventions. Firstly, recognizing the distinct valuation of emotional distress positions the imperative for public health policies that encompass mental health considerations. Comprehensive smoke-free legislation that not only safeguards physical health but also attends to the emotional well-being of nonsmokers could be pivotal. Moreover, identifying emotional distress as a valued attribute can serve as an advocacy tool, underscoring the urgency of strategies targeting the mitigation of SHS exposure.

Incorporating these insights into the broader health policy landscape fosters the construction of more holistic policy frameworks. The elevation of mental health considerations within the context of SHS exposure aligns with the growing societal recognition of mental well-being as an integral component of public health. As the valuation of emotional distress offers novel insights into SHS-related health risks, its incorporation into policy narratives can invigorate the discourse surrounding smoke-free initiatives.

The heightened valuation of emotional distress among American respondents in the context of SHS health risks affords a compelling perspective on the interplay between mental health, societal consciousness, and policy interventions. The convergence of these factors underscores the significance of crafting policy measures that holistically address nonsmokers’ physical and emotional well-being. Integrating mental health considerations into the fabric of smoke-free policies can cultivate a broader and more inclusive approach to public health.

Even with the valuable insights derived from our study concerning the valuation of secondhand smoke (SHS) health risks among American and British nonsmokers, certain limitations merit consideration in interpreting our findings. While informative, the assumptions underpinning our valuation results do not comprehensively account for underlying differentials in healthcare access, encompassing variables such as user costs and service choice. Moreover, the intricate fabric of legislative frameworks, spanning the nature of jurisdiction and the coexistence of federal and state laws in the U.S., and the nuanced applications within the unitary system of the U.K. across its constituent nations, introduce complexities that could potentially influence the generalizability of certain conclusions based on the evidence obtained.

This study stands as a pioneering endeavor, offering unprecedented insights into the valuation of secondhand smoke health risks from the vantage point of nonsmokers. The revealed outcomes augur the impetus for further scholarly inquiry. This research direction holds salience in informing the trajectory of future smoke-free legislation, particularly within the evolving landscape following the COVID-19 pandemic. Delving deeper into the dynamics of political and legislative systems, particularly concerning their potential to facilitate health objectives and universal coverage, presents a fertile terrain for subsequent investigation.

Moreover, the discernible divergence in willingness to pay (WTP) between U.S. residents and counterparts from other regions engenders intriguing questions regarding the intricate interplay between perceptions of personal freedom and the attendant financial contributions. The formulation of future investigations aimed at unraveling these complexities, examining their implications for public health policies, and dissecting the societal constructs that shape such valuation patterns, holds the promise of yielding a more profound understanding of the intricate tapestry surrounding this topic.

While acknowledging the limitations of our current study, our findings serve as a catalyst for further scholarly engagement. Exploring uncharted territories in healthcare access, legislative systems, and societal perceptions can enrich our comprehension of the valuation of secondhand smoke health risks and their ramifications. These potential avenues of research can furnish critical insights that will significantly contribute to the refinement of public health strategies, thereby navigating the complexities of a post-COVID-19 era and advancing the discourse surrounding smoke-free legislation.

## A Appendix

See Tables 12 and 13.

**Table 12:** Summary statistics of choice experiment models (U.S.)

Utility function variables	Description	Mean	Std. Dev.	Min.	Max.	Obs.
Stroke risk	Continuous	17.38	5.566	10	25	8656
Lung cancer risk	Continuous	22.51	11.758	8	38	8656
Coronary heart disease risk	Continuous	20.33	11.473	5	35	8656
Emotional distress risk	Dummy	0.5	0.500	0	1	8656
Health insurance premium	Continuous	14.900	11.224	0	30	8656

**Table 13:** Summary statistics of choice experiment models (U.K.)

Utility function variables	Description	Mean	Std. Dev	Min	Max	Obs.
Stroke risk	Continuous	17.34	5.587	10	25	8864
Lung cancer risk	Continuous	22.67	11.724	8	38	8864
Coronary heart disease risk	Continuous	20.31	11.453	5	35	8864
Emotional distress risk	Dummy	0.5	0.500	0	1	8656
Tax	Continuous	14.900	11.224	0	30	8864

We present other summary statistics not used in the models in Table 14.

**Table 14:** Other summary statistics

Variable	Description	Mean (Std. Dev.)		
		Pooled	US	UK
SHS frequency	= 1 if ETS exposure is 4 to 7 times a week	0.58 (0.493)	0.45 (0.498)	0.72 (0.450)
SHS COVID-19	= 1 if SHS exposure increased during COVID-19 lockdown			0.13 (0.334)
Consume alcohol	= 1 if respondent consumes alcohol	0.68 (0.466)	0.56 (0.497)	0.80 (0.401)
Alcohol quantity	= Average glasses of alcohol per week	2.7 (4.278)	2.1 (4.235)	3.4 (4.236)
Age	= Average age in years	32.2 (11.215)	31.4 (10.511)	32.1 (11.839)
High school diploma	= 1 if yes	0.18 (0.386)	0.21 (0.411)	0.15 (0.356)
College diploma	= 1 if yes	0.44 (0.497)	0.49 (0.500)	0.40 (0.492)
University degree	= 1 if yes	0.24 (0.424)	0.24 (0.427)	0.23 (0.422)
Other educ	= 1 if yes	0.14 (0.349)	0.06 (0.253)	0.21 (0.411)
Children	= 1 if children are living in household	0.33 (0.469)	0.33 (0.472)	0.32 (0.467)
Pet	= 1 if respondent owns a pet	0.49 (0.500)	0.55 (0.498)	0.43 (0.496)
Hhold size	= Household size	3.2 (1.446)	3.1 (1.385)	3.2 (1.505)
Employment	= 1 if in full employment	0.71 (0.454)	0.73 (0.444)	0.69 (0.463)
Apartment	= 1 if living in own apartment	0.54 (0.498)	0.55 (0.498)	0.54 (0.499)
Env group	= 1 if respondent belongs to an environmental group	0.06 (0.231)	0.08 (0.267)	0.04 (0.186)
Race	= 1 if white	0.72 (0.449)	0.63 (0.629)	0.81 (0.393)

### Robustness checks

See Table 15.

**Table 15:** Model estimates with interactions

Variable	Pooled Data		US		UK	
	OLS	Tobit	OLS	Tobit	OLS	Tobit
Dummy$$^{\dagger }$$	0.504*** (0.136)	0.500*** (0.133)				
Male	0.912*** (0.272)	0.895*** (0.273)	0.532 (0.470)	0.462 (0.481)	1.323*** (0.339)	1.325*** (0.318)
Exposure place$$\times$$distress	0.554** (0.233)	0.559** (0.222)	0.378 (0.395)	0.379 (0.362)	0.807*** (0.301)	0.809*** (0.275)
Income: $1100-$5600	0.366** (0.183)	0.353* (0.197)	0.454 (0.358)	0.384 (0.388)	0.238 (0.212)	0.248 (0.213)
Income: $5601-$10100	0.531 (0.425)	0.497 (0.379)	0.598* (0.257)	0.517 (0.501)	1.473*** (0.230)	1.484 (1.473)
Income: More than $10100	1.370*** (0.357)	1.358*** (0.488)	1.746*** (0.470)	1.689** (0.678)	0.631 (0.544)	0.643 (0.672)
Living with partner	− 0.333** (0.131)	− 0.339*** (0.130)	− 0.486*** (0.196)	− 0.568*** (0.198)	− 0.127 (0.179)	− 0.118 (0.167)
Partner smokes	1.081*** (0.257)	1.087*** (0.286)	1.109** (0.550)	1.133** (0.508)	1.172*** (0.247)	1.165*** (0.331)
Health status	0.067 (0.126)	0.070 (0.124)	− 0.244 (0.194)	− 0.230 (0.189)	0.375** (0.160)	0.367** (0.159)
SHS knowledge: Good	0.043 (0.140)	0.032 (0.142)	0.350 (0.225)	0.345 (0.228)	− 0.201 (0.180)	− 0.214 (0.173)
SHS knowledge: Excellent	0.106 (0.203)	0.086 (0.195)	0.579* (0.305)	0.560* (0.290)	− 0.302 (0.251)	− 0.316 (0.259)
Male$$\times$$income: $1100-$5600	− 0.349 (0.309)	− 0.338 (0.309)	− 0.142 (0.518)	− 0.074 (0.533)	− 0.625 (0.386)	− 0.635* (0.372)
Male$$\times$$income: $5601-$10100	− 0.156 (0.572)	− 0.132 (0.507)	− 0.133 (0.711)	0.212 (0.673)	− 0.363 (1.220)	− 0.376 (1.715)
Male$$\times$$income: More than $10100	− 0.948 (0.608)	− 0.929 (0.646)	− 0.787 (0.725)	− 0.714 (0.861)	− 2.348*** (0.770)	− 2.361* (1.310)
Constant	3.247*** (0.222)	3.266*** (0.238)	3.857*** (0.402)	3.920*** (0.426)	3.034*** (0.276)	3.036*** (0.282)
Sigma		2.604 (0.138)		2.927 (0.221)		2.113 (0.157)
Log-likelihood		− 1376.7218		− 712.1583		− 650.842
R-squared	0.125	0.033	0.096	0.024	0.155	0.045
Obs.	727	727	364	364	363	363

***$p<$0.01, **$p<$0.05, *$p<$0.10. Standard errors (in parentheses) are robust. $\dagger$ =1 if US and 0 if UK. The tobit model is left and right-censored

The proportion of zero responses presented in Table 16 reveals that approximately 33% of respondents from the United States and 34% from the United Kingdom provided zero values. This phenomenon is interpreted as a form of protest, as a significant number of participants expressed an unwillingness to pay for others' risky behaviors. For those respondents who assigned a positive value, an inquiry was made regarding their level of certainty in actualizing the stated payment. Notably, 13.49% of U.S. respondents and 15.52% of U.K. respondents indicated uncertainty, while 22.37% and 27.26%, respectively, expressed assurance, and 14.97% and 22.74%, respectively, conveyed a high level of confidence in their willingness to pay. Furthermore, within the category of respondents expressing a high level of certainty, U.S. participants demonstrated a willingness to pay $864.07, in contrast to the $131 indicated by their U.K. counterparts.

**Table 16:** WTP by segment (U.S. & U.K.)

	U.S.		U.K.	
	Proportion (%)	Mean WTP	Proportion (%)	Mean WTP
Protest	32.72	0	34.48	0
Not sure	13.49	$538.38 (1861.92)	15.52	$405.51 (2949.60)
Sure	22.37	$288.85 (667.09)	27.26	$242.70 (731.84)
Very sure	14.97	$864.07 (3323.28)	22.74	$131 (246.56)
No response*	16.45	$512.71 (1306.85)	–	–

Standard deviations are in parentheses. *No response is a segment of the sample that did not indicate how certain they were to pay the stated WTP
